# Gut-Mediated Systemic Toxicity of Micro- and Nanoplastics: Nanoscale Biointerface Properties, Microbiota-Metabolite Crosstalk, and Evidence Across Gut-Organ Axes

**DOI:** 10.3390/nano16150923

**Published:** 2026-07-27

**Authors:** Mi Wang, Lulu Wang, Na Li, Meizhen Wang, Kun Lu

**Affiliations:** Zhejiang Key Laboratory of Solid Waste Pollution Control and Resource Utilization, School of Environmental Science and Engineering, Zhejiang Gongshang University, Hangzhou 310018, China

**Keywords:** microplastics, nanoplastics, gut-organ axis, nano-biointerface, gut microbiota, metabolomics, intestinal barrier, systemic toxicity

## Abstract

Microplastics and nanoplastics (MNPs) have been recognized as ubiquitous emerging global pollutants, which are extensively detectable in diverse environmental media and food matrices. Increasing evidence indicates that the intestine is a primary target of orally ingested MNPs and a critical initiating hub for systemic toxicity. Once ingested orally, MNPs can interact with the intestinal mucus layer and epithelial barrier, induce gut microbiota dysbiosis, remodel bile acid and short-chain fatty acid metabolism, and activate oxidative stress, inflammation, apoptosis, and immune imbalance. These gut-derived disturbances may subsequently propagate adverse signals to distal organs through the gut-liver, gut-brain, gut-kidney, gut-lung, gut-reproductive, and gut-mammary axes. Intestinal barrier dysfunction, endotoxin translocation, abnormal microbial metabolites, and microbiota-derived immune signals constitute common mediating pathways linking local intestinal injury to multi-organ toxicity. In addition, smaller particle size, surface oxidation, environmental aging, bio-corona/plastisphere formation, and co-exposure with other contaminants can further modulate the intensity and specificity of gut-organ axis disruption. Prior reviews are limited to separate analyses of single-organ toxicity or isolated gut-organ pathways. To fill this gap, this work synthesizes contemporary mechanistic and experimental evidence to establish a gut-initiated systemic toxicology framework for MNPs. We differentiate direct particle translocation from gut-derived indirect signaling, evaluate the varying robustness of supporting evidence for each gut-organ axis, and underscore nanoscale biointerface properties as key modulators of MNPs systemic toxic potency.

## 1. Introduction

The large-scale production and widespread use of plastic products have promoted the development of modern society [1,2,3], but have also led to the persistent accumulation of plastic waste in the environment, where it can further fragment into microplastics (MPs) and nanoplastics (NPs) [4,5]. Current studies generally define plastic particles smaller than 5 mm as MPs, whereas smaller particles, usually less than 1 μm, are commonly classified as NPs. The World Health Organization has noted that the main routes of human exposure to MPs and NPs include dietary intake and inhalation [6,7]. Food, water, and air together constitute a continuous, low-dose, and long-term exposure background [8,9]. However, substantial uncertainties remain regarding human exposure levels, *in vivo* fate, and health risks [10,11,12].

Compared with conventional chemical pollutants, the health risks of microplastics and nanoplastics (MNPs) are more complex [13]. On the one hand, MNPs are characterized by small particle size, large specific surface area, and abundant surface functional groups, which enable them to directly interact with the intestinal mucus layer, cell membranes, mitochondria, and immune cells [14,15,16]. These interactions are particularly important for NPs, whose nanoscale dimensions may facilitate trans-epithelial transport, cellular uptake, organelle stress, and systemic distribution. In addition, MNPs surfaces can adsorb organic contaminants, heavy metals, as well as pathogen-associated molecular patterns, which further modulates the bioavailability and toxicokinetic profiles of co-occurring pollutants within biological organisms [13,17,18,19,20]. Accordingly, MNPs function not only as inherent pollutants but also as effective carriers of exogenous contaminants. Their toxic effects are far beyond simple linear additivity, instead presenting as a pathological cascade involving intestinal barrier impairment, inflammatory exacerbation, and metabolic dysregulation.

In recent years, the concept of gut-organ axis has offered an innovative holistic framework to decipher MNPs-triggered systemic toxicity [21,22]. The intestine serves not only as a key organ for nutrient absorption, but also as the body’s largest immune and microbial ecological interface [23]. Upon entry into the organism, MNPs initially interact with the intestinal mucus layer, resident microbiota, and epithelial cells within the intestinal lumen [24]. Intestinal microecological dysbiosis suppresses the expression of tight junction proteins and elevates intestinal barrier permeability. Subsequently, lipopolysaccharide (LPS), bile acids, short-chain fatty acids (SCFAs), tryptophan metabolites, arachidonic acid derivatives, and inflammatory mediators can target distal organs *via* the portal vein, systemic circulation, and neuroendocrine pathways, ultimately initiating pathological responses across the gut-liver, gut-brain, gut-kidney, gut-lung, and gut-reproductive axes. Therefore, the central question is no longer whether MNPs can damage individual organs, but how intestinal injury is translated into systemic toxicity through interorgan communication.

Research advances in MNPs-mediated disruption of the gut-organ axis have gradually shifted from preliminary phenotypic observation to in-depth mechanistic exploration, with a core focus on the microbiota, metabolite, barrier, immunity, and cell death cascade [25,26]. Over the last two to three years, emerging methodologies including multi-omics profiling, fecal microbiota transplantation (FMT), antibiotic-driven microbiota depletion, tissue tracing, and organoid culture have allowed researchers to explicitly validate the mediating function of gut microbiota in remote organ damage [24]. Even so, human epidemiological evidence, realistic real-world exposure simulation, and unified analytical standards remain severely lacking [27]. Herein, it is worth noting that progress has been uneven across gut-organ axes. The gut-liver axis is supported by multiple intervention and multi-omics studies, whereas the gut-brain axis has emerging but heterogeneous mechanistic evidence. By contrast, gut-kidney, gut-lung, gut-reproductive, and gut-mammary mechanisms remain based largely on limited preclinical, co-exposure, or single-study observations. The following sections therefore evaluate each axis according to its evidential maturity. Actually, several recent reviews and perspectives have addressed individual components of this topic (Table 1), including microplastic-associated gut-brain signaling and neurovascular outcomes, MNPs-related liver disease, human intestinal exposure and analytical standardization, reproductive toxicity, and human digestive, reproductive, and respiratory health outcomes. These studies provide important organ-specific, microbiome-centered, or evidence-assessment perspectives; however, cross-axis comparison and a shared mechanistic sequence from intestinal initiation to distal-organ injury were not their primary focus. However, rarely do prior works organize these scattered pathological alterations into a time-ordered causal chain rooted in primary intestinal impairment. Furthermore, few publications construct a clear hierarchical and sequential toxicological logic centered on gut-derived initiation to collectively explain the wide range of subsequent multi-organ injuries induced by MNPs. In addition, earlier summaries almost never identify and formalize the three core shared transduction cascades (immune–inflammatory, metabolic, and neural–endocrine pathways) that act as universal mediators transmitting gut dysfunction to remote organs across all gut-organ communication axes. In this context, a systematic review addressing the perturbing effects of MNPs on gut–organ axes and their molecular mechanisms is of great theoretical significance and practical implication. Taken together, this review aims to (1) summarize current evidence for MNPs-induced disruption of major gut-organ axes, (2) distinguish causally validated mechanisms from correlative observations, (3) identify shared molecular events across different axes, and (4) highlight how nanoscale particle properties and environmental transformation modulate systemic toxicity.

## 2. Literature Search Strategy and Evidence Classification

A literature search was conducted using Web of Science, PubMed, Scopus, and Google Scholar to identify studies related to microplastics, nanoplastics, and gut-organ axis toxicity. The search terms included “microplastics”, “nanoplastics”, “gut-organ axis”, “gut-liver axis”, “gut-brain axis”, “gut-kidney axis”, “gut-lung axis”, “gut microbiota”, “intestinal barrier”, “metabolomics”, “oxidative stress”, “inflammation”, “ferroptosis”, and “pyroptosis”. Priority was given to studies published in the past five years (2021–2026) that provided mechanistic evidence linking intestinal injury, microbiota dysbiosis, metabolite remodeling, and distal organ toxicity (Table 2).

Studies were included when they reported at least one of the following aspects: particle characterization, intestinal barrier dysfunction, gut microbiota alterations, metabolomic changes, distal organ injury, or causal validation using fecal microbiota transplantation, antibiotic-mediated microbiota depletion, germ-free animals, metabolite supplementation, or pathway inhibition. Studies focusing only on environmental occurrence without biological endpoints were excluded.

## 3. The Intestine as the Initiating Hub of MNPs-Induced Systemic Toxicity

**Figure 1:**
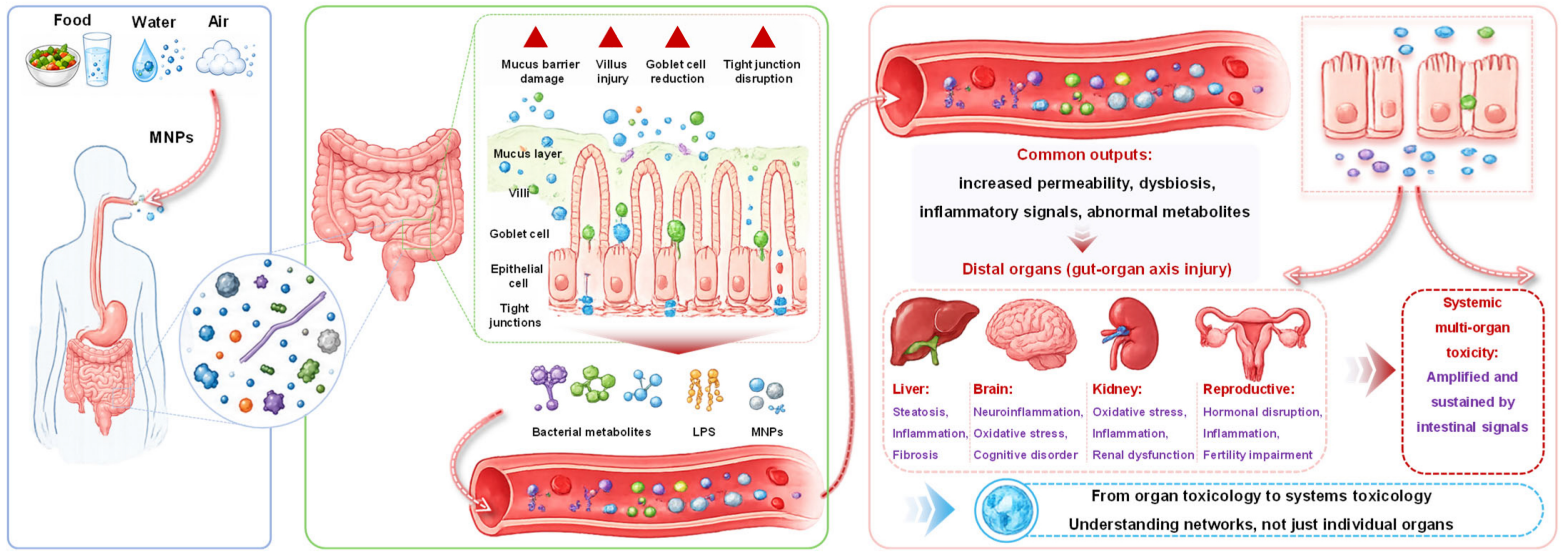
The intestine as the initiating hub of MNPs-induced systemic toxicity and its mechanistic framework. The intestine is not only the primary target organ following oral exposure to MNPs, but also a key hub for amplifying and transmitting systemic toxicity, implying that research on MNPs toxicity should shift from single-organ toxicology toward a systems toxicology framework based on a “gut-first, organ-later” paradigm. The figure was created with BioRender, PowerPoint and Adobe Illustrator.

The intestine represents the primary entry site and initiating hub for MNPs-induced toxic cascades, mainly because orally ingested particles first accumulate and interact with the gastrointestinal microenvironment [43,44]. After oral ingestion, MNPs reside within the gastrointestinal tract and engage in intricate interactions with the mucus layer, digestive juices, chyme constituents, and microbial metabolites. Accumulating evidence reveals that MNPs compromise mucus barrier integrity, trigger intestinal villus morphological injury and goblet cell loss, and suppress the expression of tight junction proteins such as occludin, claudin-1, and ZO-1. These alterations ultimately disrupt the intestinal epithelial barrier and give rise to a leaky gut phenotype [43,45,46,47]. Upon intestinal barrier dysfunction, LPS, bacterial metabolites, and intact MNPs gain enhanced permeability across the intestinal epithelium, enabling their entry into the systemic circulatory system [48,49], as illustrated in Figure 1.

Gut microbiota dysbiosis is another central event in MNPs-induced intestinal toxicity [24,50]. Both review and experimental studies have shown that MNPs reduce microbial alpha diversity, deplete beneficial genera including *Lactobacillus*, *Bifidobacterium*, and *Akkermansia*, enrich opportunistic pathogens, shift the Firmicutes/Bacteroidota ratio, and remodel metabolic pathways associated with lipid and bile acid metabolism, carbohydrate utilization, and short-chain fatty acid biosynthesis [7,51,52]. These microbial and metabolic alterations disrupt intestinal homeostasis and transform the gut from a physiological regulatory interface into a source of systemic inflammatory and metabolic signals.

Importantly, MNP-triggered intestinal inflammation is no longer restricted to local intestinal lesions. Mounting evidence shows that MNPs activate the TLR4/MyD88/NF-κB signaling axis [53], NLRP3 inflammasome assembly [54], oxidative stress, and lipid peroxidation. These events collectively establish a self-amplifying cascade characterized by reactive oxygen species (ROS) overaccumulation, mitochondrial dysfunction, pro-inflammatory cytokine secretion, and subsequent cell death [55,56,57]. Synergistically coupled with microbiota-driven metabolic reprogramming, this pathological process imposes a dual stress on distal organs via the delivery of pro-inflammatory signals and aberrant metabolites. Through systemic circulation, the portal vein, and neuroendocrine routes, these gut-derived signals may subsequently affect distal organs and contribute to multi-organ toxicity [47].

From a systems toxicology perspective, the intestine should therefore be considered not only a primary target organ of MNPs exposure, but also a critical amplifier and transmitter of systemic toxicity. Focusing exclusively on end-stage injuries in the liver, brain, kidney, lung, or reproductive organs, while neglecting the mediating roles of the intestinal barrier and gut microbiota, may fail to fully explain the mechanisms underlying low-dose, chronic, and remote organ damage induced by MNPs. A growing body of evidence lends support to a “gut-first, organ-later” conceptual framework. Within this model, compromised intestinal barrier integrity, gut microbial dysbiosis, altered metabolite profiles, and immune inflammatory activation appears to act in concert to mediate gut-organ axis dysfunction triggered by MNPs. This gradual conceptual evolution reflects a notable shift in MNP toxicology research: moving beyond traditional approaches focused on isolated organ toxicity assessments toward a more holistic, systems-based toxicology paradigm.

## 4. Disruptive Effects and Mechanisms of MNPs on the Gut-Organ Axis in Organisms

### 4.1. Disruptive Effects of MNPs on the Gut-Liver Axis and Their Mechanisms

Among the various gut-organ axes, the gut-liver axis is currently the one with the most substantial evidence and the clearest mechanistic understanding [58,59]. Anatomically and physiologically, the liver is closely connected to the intestine through the portal venous system and the enterohepatic circulation of bile acids. Nutrients, bacterial components, microbial metabolites, and intestinal inflammatory mediators can be delivered directly from the gut to the liver via the portal vein, whereas bile acids, IgA, and liver-derived metabolites can in turn regulate intestinal microbial ecology [59]. Therefore, MNPs-induced intestinal barrier dysfunction, gut microbiota dysbiosis, and metabolic disturbance can be readily transmitted to the liver, where they manifest as inflammation, lipid accumulation, bile acid metabolic abnormalities, oxidative stress, and cell death [26]. It should be critically noted that the above mechanistic chain is predominantly summarized from short-term, high-dose rodent exposure assays. Most *in vivo* studies adopt oral gavage or single/daily bolus administration far exceeding the daily human oral intake of MPs/NPs under environmental background levels. Such acute exposure regimens trigger severe intestinal epithelial erosion and sharp microbiota shifts that rarely emerge under chronic low-dose environmental exposure. Moreover, nearly all supportive data rely on mouse models; interspecies differences in intestinal mucus thickness, bile acid pool composition, and hepatic metabolic enzyme profiles create major uncertainties when extrapolating gut-liver toxic outcomes to human populations, and validated human tissue or organoid evidence to verify these cascading pathways remains extremely scarce.

An important perspective emerging from recent studies is that MNPs-induced liver injury is not solely attributable to direct particle deposition in hepatic tissue, but is also strongly mediated by gut-derived signals [26]. For example, dietary exposure to polylactic acid microplastics (PLA MNPs) can alter the gut microbiota, intestinal and serum metabolomes, induce hepatic transcriptomic changes, and lead to hepatotoxicity [60]. In contrast, polystyrene microplastics (PS MPs) disrupted intestinal homeostasis by disturbing the gut microbiota, inducing colonic inflammation, and damaging the intestinal barrier. These intestinal alterations subsequently triggered pan-hepatic and even systemic inflammation through the gut-liver axis and induced hepatic metabolic disorders. This conclusion was validated through gut microbiota depletion and FMT. Although the results indicated that PS MPs-induced liver fibrosis was independent of the gut microbiota, the study further showed that epigallocatechin-3-gallate (EGCG) increased the abundance of probiotics and effectively suppressed PS MPs-induced colonic inflammation, thereby alleviating systemic and hepatic inflammation, hepatic metabolic disorders, and fibrosis induced by PS MPs [61]. A critical unresolved contradiction exists within microbiota-mediated toxic phenotypes, that is, the identical PS MPs exposure triggers microbiota-dependent hepatic inflammation but microbiota-independent liver fibrosis, which suggests multiple parallel, uncoupled gut-liver signaling branches coexist yet remain poorly dissected. Current FMT and antibiotic depletion experiments cannot fully distinguish direct particle toxicity from indirect microbial metabolite effects, as broad-spectrum antibiotic treatment itself disturbs intestinal bile acid cycling and epithelial permeability as independent confounding factors. In addition, few studies control for copurified plastic additives, plastic leachates, or microbial endotoxin loads during FMT, which may artificially amplify or mask the genuine gut-mediating effects of MNPs.

In addition to microbiota dysbiosis and barrier disruption, changes in the biological identity of MNPs within the intestinal microenvironment may also influence liver toxicity. MNPs can interact with intestinal components to form bio-coronas and plastispheres, which modify their surface properties, alter their interaction with immune cells, and promote macrophage internalization [26]. Under conditions of increased intestinal permeability, such as liver cirrhosis or inflammatory intestinal injury, the translocation of particles and particle-associated substances through the portal circulation may be further enhanced. These findings suggest that MNPs should not be regarded as inert physical particles, but rather as dynamic particulate entities with combined biological, chemical, and carrier-related activities. Current descriptions of intestinal bio-corona formation remain highly simplified. Existing experiments mostly construct bio-coronas *via* single intestinal protein or metabolite incubation in vitro, failing to replicate the complex dynamic mixture of mucus glycoproteins, bile acid micelles, digestive enzymes, and live commensal bacteria within the intact intestinal lumen. The reversible, time-dependent remodeling of bio-corona during gastrointestinal transit and enterohepatic circulation is rarely tracked. Furthermore, it remains unclear whether plastisphere aggregation facilitates particle translocation or conversely restricts intestinal epithelial uptake, with conflicting *in vitro* and *in vivo* observations lacking unified mechanistic explanations.

Bile acid homeostasis disruption represents a central mechanism linking MNPs-induced intestinal injury to hepatic dysfunction [26]. Wen et al. reported that MPs at environmentally realistic concentrations provoke cholestasis and bile acid metabolic dysregulation through a well-defined four-step gut-liver circuit [62]. These findings indicate that MNPs exposure not only induces inflammation and oxidative stress, but also interferes with gut microbial bile acid transformation, hepatic bile acid synthesis and transport, and enterohepatic recirculation. Such perturbations amplify initial intestinal lesions into pervasive systemic metabolic dyshomeostasis. Accumulating data further confirm that PS NPs efficiently cross the intestinal barrier and trigger sex-dependent toxicity modulated by bile acids and gut microbiota [63]. Using rhodamine B-labeled PS NPs, a previous study found that approximately 60% of orally administered 50 mg/kg PS NPs (∼70 nm diameter and −30.2 mV zeta potential) were translocated across the intestinal epithelium within 3 h, with most particles subsequently retained in the liver and cleared via biliary excretion. Surface-adsorbed bile acids and the apical sodium-dependent bile acid transporter facilitated the intestinal uptake of PS NPs. In hepatocytes, PS NPs disrupted lysosomal biogenesis and suppressed CYP7A1 degradation, thereby accelerating bile acid biosynthesis. Additionally, PS NPs exposure reduced *Lactobacillus* abundance and increased *Enterobacteriaceae*, enhancing susceptibility to intestinal inflammation. The stronger toxicity observed in male mice was associated with higher intestinal apical sodium-dependent bile acid transporter (ASBT) expression, suggesting that sex-dependent bile acid transport may modulate NPs uptake and toxicity [63]. The sex-dependent toxicity driven by ASBT expression is only validated for narrow-sized, negatively charged PS NPs. Whether this sex-specific bile acid transport rule applies to positively charged particles, biodegradable PLA fragments, or aged environmental NPs remains unconfirmed. In addition, rapid short-term translocation assays (3 h post-administration) cannot reflect long-term cumulative particle retention and chronic bile acid disturbance under repeated low-dose exposure; acute bolus uptake data may overestimate the translocation efficiency of MNPs under real daily ingestion scenarios.

Lipid metabolic disorder is another hallmark of gut-liver axis dysfunction induced by MNPs [22,64]. Current evidence reveals that MNPs exposure commonly induces elevated triglyceride contents, excessive lipid droplet deposition, dysregulated fatty acid metabolic pathways, and heightened inflammatory mediator expression [65]. Across fish and mammalian experimental models, compositional shifts in the gut microbiota are tightly linked to hepatic immune activation and dysregulated transcription of bile acid metabolism-associated genes. This indicates that intestinal microecological perturbation can reshape hepatic lipid homeostasis via interorgan metabolic crosstalk [66,67]. As a typical example, PLA MPs perturb gut microbial community structure, consequently raising uric acid levels in both plasma and liver tissues. The accumulated uric acid upregulates hepatic *Hsd17b13* transcription, which in turn drives triglyceride overaccumulation, hepatic inflammation, and fibrotic progression. Notably, gut microbiota depletion effectively mitigates PLA microplastic-elicited liver damage in mice. PLA MPs alone fail to directly upregulate HSD17B13 or induce lipid deposition in HepG2 cells, whereas uric acid exposure recapitulates these pathological phenotypes, underscoring uric acid as a critical mediator of lipid metabolic dysfunction. Collectively, these results establish that PLA MPs-related hepatotoxicity is mediated by gut microbiota-derived uric acid overproduction, and further validate that biodegradable MPs can likewise induce systemic toxic disturbance [65]. Therefore, the microbiota–bile acid/uric acid–lipid metabolism–inflammation cascade has become one of the most informative mechanistic frameworks for understanding MNPs-induced gut-liver axis injury.

Although both MPs and NPs can induce hepatic damage through the gut-liver axis, their dominant modes of action may differ [47,52,68,69]. MPs, owing to their relatively larger size, are more likely to remain within the intestinal lumen, mucus layer, or epithelial surface. Their hepatotoxicity is therefore mainly driven by gut-initiated indirect mechanisms. By damaging the mucus barrier, reducing tight junction proteins such as occludin, claudin-1, and ZO-1, and inducing gut microbiota dysbiosis, MPs promote the portal delivery of LPS, abnormal metabolites, and inflammatory mediators to the liver. These gut-derived inputs subsequently trigger hepatic inflammation, oxidative stress, lipid accumulation, metabolic disorder, and fibrotic responses. Thus, MPs-induced hepatotoxicity generally follows a cascade of intestinal barrier impairment, microbiota dysbiosis, abnormal portal venous input, and hepatic pathological response.

By comparison, NPs possess smaller dimensions, larger specific surface area, and higher surface reactivity, allowing them to more efficiently cross the intestinal barrier and enter systemic circulation. After translocation, NPs may be taken up by hepatocytes, Kupffer cells, and other resident hepatic cells. Therefore, NPs-induced gut-liver axis injury involves both indirect gut-derived signaling and direct hepatic cellular damage. Once deposited in the liver, NPs can disrupt lysosomal, mitochondrial, and endoplasmic reticulum homeostasis, interfere with bile acid synthesis and trafficking, enhance oxidative stress and inflammasome activation, and trigger programmed cell death, including pyroptosis and ferroptosis. Compared with MPs, NPs may therefore induce more extensive gut-liver toxicity through the combined effects of intestinal barrier penetration, hepatic accumulation, and intracellular organelle injury.

In realistic environmental and dietary exposure scenarios, MPs and NPs rarely occur as isolated, pristine particles. MPs may generate nanosized fragments during environmental weathering, gastrointestinal digestion, and mechanical abrasion. Furthermore, NPs can adsorb coexisting pollutants, including polycyclic aromatic hydrocarbons, per- and polyfluoroalkyl substances, and heavy metals, thereby acting as carriers that deliver these contaminants to the intestine and liver. Consequently, MPs-induced gut-liver axis injury under real-world conditions is likely to result from the combined effects of MP-mediated disruption of intestinal ecology and barrier integrity, NPs-mediated trans-barrier transport and hepatic intracellular injury, and the amplification of co-contaminant toxicity.

**Figure 2:**
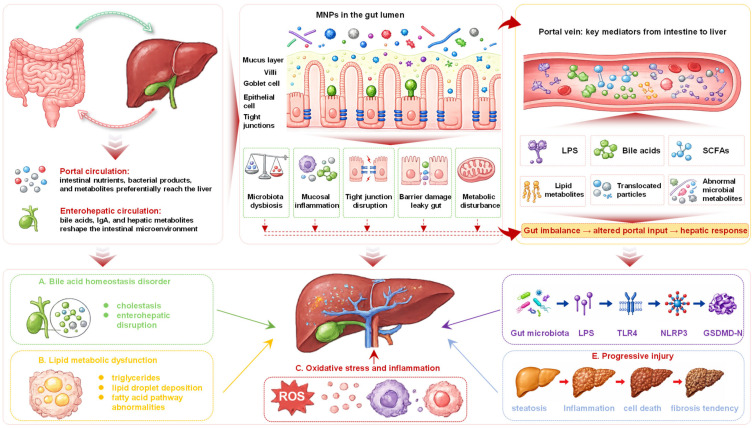
MPs/NPs disrupt the gut-liver axis. After oral exposure, MNPs disturb gut microbiota, induce mucosal inflammation, disrupt tight junction proteins, impair intestinal barrier integrity, and alter host-microbial metabolism. These intestinal events reshape portal venous input by increasing the delivery of LPS, bile acids, SCFAs, lipid metabolites, translocated particles, and abnormal microbial metabolites to the liver. In response, the liver develops bile acid metabolic disorder, lipid accumulation, oxidative stress, inflammation, pyroptosis, and fibrotic tendency, ultimately contributing to hepatic dysfunction and systemic toxicity. The figure was created with BioRender, PowerPoint and Adobe Illustrator.

Taken together, current evidence suggests that MNPs-induced gut-liver axis disruption follows a relatively clear pathological sequence. As illustrated in Figure 2, first, MNPs impair intestinal barrier integrity, induce mucosal inflammation, and remodel gut microbiota. Second, these intestinal alterations modify portal venous input by increasing the transport of LPS, bile acids, SCFAs, lipid metabolites, inflammatory mediators, and translocated particles. Finally, these gut-derived signals manifest in the liver as bile acid dysregulation, lipid metabolic disorder, oxidative stress, inflammatory activation, programmed cell death, and fibrotic progression. Therefore, the gut-liver axis should not be considered a secondary consequence of MNPs-induced hepatotoxicity, but rather one of its core mechanistic pathways. Notably, nearly all existing mechanistic studies adopt single pristine polymer particle treatments, ignoring synergistic or antagonistic toxic interactions between plastic fragments and adsorbed organic/inorganic pollutants. Few *in vivo* trials simulate long-term sequential weathering, gastrointestinal fragmentation, and co-contaminant desorption within the gut lumen. The additive or amplified gut-liver toxicity hypothesized here remains largely theoretical, with insufficient quantitative in vivo evidence to disentangle particle carrier effects from independent toxic effects of adsorbed chemical pollutants.

### 4.2. Disruptive Effects of MPs and NPs on the Gut-Brain Axis and Their Mechanisms

The gut-brain axis has become one of the fastest-growing research directions in the field of MNPs in recent years [25,70]. Traditional neurotoxicology has largely emphasized the direct neurotoxicity of particles after crossing the blood-brain barrier. However, recent studies suggest that after entering the body through oral exposure, MNPs first alter the gut microecology, barrier integrity, and immune status, and subsequently affect brain function through neural, immune, and endocrine networks [70]. Therefore, MNPs-induced neurotoxicity does not necessarily depend solely on extensive particle deposition in brain tissue. Gut-derived inflammation, metabolic disturbance, and neurotransmitter imbalance may also be sufficient to trigger neuroimmune activation and behavioral abnormalities.

Representative studies have begun to provide mechanistic evidence for this gut-mediated neurotoxicity. In juvenile mice, exposure to PS NPs induced pronounced neurotoxic effects [70]. Proteomic analysis and subsequent validation revealed that disruption of brain protein homeostasis, mediated by lysosomal and proteasomal dysfunction, was a key mechanism underlying PS NPs-induced neurotoxicity in juvenile mice. Through 16S rDNA analysis and gut microbiota transplantation, it was found that the gut microbiota played an indirect role in PS NPs-induced neurotoxicity. High-dimensional mediation analysis then quantified, for the first time, the average indirect effect of the gut microbiota as 39.20%, while key taxa such as *Eubacterium coprostanoligenes* may serve as indicator species of microbiota dysbiosis. In addition, they also identified trehalose as a mediator linking the gut microbiota with nervous system function and emphasized that trehalose supplementation can reshape brain protein homeostasis, thereby alleviating neurotoxicity in juvenile mice [70].

Interestingly, NPs were efficiently phagocytosed by macrophages, leading to lysosomal damage [71]. NPs were efficiently phagocytosed by intestinal macrophages, leading to lysosomal damage and the emergence of a macrophage subpopulation characterized by high IL-1β expression. These intestinal macrophages released IL-1β into systemic circulation, which subsequently activated brain microglia and promoted Th17 cell differentiation in the brain. These immune alterations were associated with impaired learning and memory functions. This study provides important evidence that intestinal immune activation can transmit neuroinflammatory signals to the brain, thereby linking local NP-induced intestinal injury with distal cognitive impairment [71]. This immune cascade relies on circulating IL-1β as the core peripheral messenger, yet the study fails to exclude contributions from other gut-derived pro-inflammatory mediators (TNF-α, LPS, tryptophan metabolites) that simultaneously rise upon intestinal macrophage activation. Furthermore, the pathway by which peripheral IL-1β crosses the blood–brain barrier to trigger microglial polarization is not validated via targeted receptor blockade or endothelial permeability assays. Whether vagus nerve signaling synergizes with systemic cytokine circulation to deliver neuroinflammatory signals remains uncharacterized, leaving the immune transmission chain incomplete.

Starch-based MPs have also attracted attention because of their potential degradation into smaller particles and bioactive metabolites in the gastrointestinal tract. A previous study showed that 90-day chronic exposure to environmentally relevant doses of starch-based MPs caused intestinal structural alterations, gut microbiota dysbiosis, and fatty acid metabolic disorder in mice [72]. Since gut microbial imbalance and lipid metabolic disturbance are closely associated with neurotoxicity [73], these findings raised concern that starch-based MPs may influence central nervous system function through the gut–brain axis. Other MPs and NPs, such as PLA and PS and polyethylene terephthalate, can induce neurotoxicity by disrupting the gut-brain axis and may increase the risk of neurodegenerative diseases [50,74,75]. Moreover, starch-based MPs may degrade in the gastrointestinal tract and release smaller nanoparticles, namely starch-based NPs (SB NPs), and SCFAs. These products may cross the blood-brain barrier and affect neuroinflammation and amyloid protein formation in the brain. However, whether SB NPs influence the central nervous system by modulating the gut microbiota and thereby increase the risk of neurodegenerative diseases remains unclear, representing a critical toxicological knowledge gap. To address these issues, Deng et al. carried out a study, where mice were exposed to food-relevant concentrations of SB NPs for 180 days to evaluate their risk of Alzheimer’s disease (AD) [21]. Their results showed the presence of SB NPs in brain tissues. Meanwhile, the mice exhibited significantly impaired motor ability, learning, and memory, along with increased levels of Aβ-42 protein in the brain, indicating that SB NPs have a strong potential to promote AD-like pathology. Integrated multi-omics analysis further revealed that SB NPs drove the expansion of bacterial taxa and metabolic pathways associated with SCFAs production. The resulting excessive accumulation of SCFAs, together with the entry of SB NPs into the circulatory system and their enrichment in brain tissue, disrupted fatty acid homeostasis and triggered neuroinflammation, ultimately increasing AD risk. These findings indicate that chronic exposure to SB NPs may promote AD-like pathological features in experimental models through gut-brain axis disruption [21]. However, its relevance to human neurodegenerative disease remains to be established.

**Figure 3:**
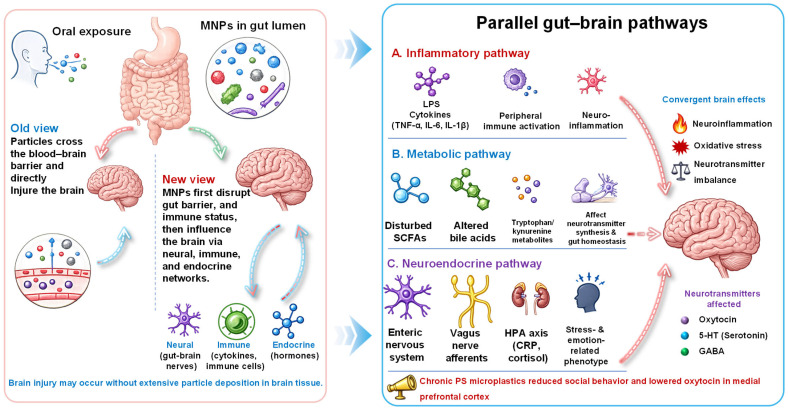
MPs/NPs disrupt the gut-brain axis. After oral exposure, MNPs disturb intestinal homeostasis by increasing gut permeability, inducing inflammation, reshaping gut microbiota, and altering microbial metabolism. These intestinal changes activate three major gut-brain communication routes: immune-inflammatory signaling mediated by LPS, IL-1β, and cytokines; metabolic signaling involving SCFAs, bile acids, fatty acids, and tryptophan/kynurenine metabolites; and neural-endocrine signaling involving the enteric nervous system, vagus nerve, and HPA axis. These pathways collectively promote neuroinflammation, oxidative stress, neurotransmitter imbalance, and behavioral alterations. The figure was created with BioRender, PowerPoint and Adobe Illustrator.

Based on the present studies, three partially overlapping candidate routes can be provisionally organized (Figure 3). The first is the immune-inflammatory route. Intestinal barrier dysfunction facilitates the systemic entry of LPS, IL-1β, and other pro-inflammatory mediators, which may activate peripheral immunity and promote neuroinflammation through microglial activation and brain immune cell remodeling [76]. The second is the metabolic route. MNPs-induced gut microbiota dysbiosis can disturb SCFAs, bile acids, fatty acids, and tryptophan/kynurenine pathway metabolites, thereby affecting neurotransmitter biosynthesis, glial homeostasis, and neuronal function [77]. The third is the neural and endocrine route. The enteric nervous system, vagal afferent signaling, and hypothalamic–pituitary–adrenal axis may jointly regulate stress-related, emotional, and cognitive phenotypes [78]. Collectively, these three interconnected signaling pathways suggest that MNPs-elicited neurotoxicity represents a whole-body pathological process marked by compromised intestinal barrier function, reshaped microbiota-metabolite profiles, widespread immune activation, and subsequent functional deficits within the central nervous system.

It is important to emphasize that particle size and surface properties can significantly influence the strength of gut-brain axis effects [79]. Smaller particles, especially NPs, are generally more likely to cross the intestinal barrier, enter systemic circulation, and interact with immune cells or neural tissues, thereby producing stronger inflammatory and behavioral effects [71]. In addition, oxidized or aged plastics may show enhanced neurotoxicity due to increased surface functional groups and stronger pollutant adsorption capacity [80,81]. For example, weathered microplastics (WMPs) generated under environmentally mimetic conditions exhibited rougher surfaces, reduced molecular weight, and structural alterations compared with virgin microplastics. Proteomic analysis showed stronger activation of immune- and neurodegeneration-related pathways in the WMPs group. Consistently, human microglial HMC-3 cells displayed more severe inflammatory responses to WMPs than to virgin microplastics [81]. These results show that WMPs are a more profound inflammatory factor than VMPs, implying environmental transformation can substantially modify the neurotoxic potential of MNPs.

Overall, MNPs-induced gut-brain axis disruption can be understood as a systemic response that is initiated in the gut and manifested in the brain. Current evidence supports the involvement of intestinal barrier dysfunction, gut microbiota dysbiosis, immune inflammatory activation, metabolic remodeling, and, in some cases, direct particle entry into brain tissue. Future studies should further distinguish which neurobehavioral outcomes are primarily mediated by gut-derived signals and which are more closely associated with direct particle translocation into the brain. In addition, integrated analyses of LPS, SCFAs, secondary bile acids, tryptophan metabolites, oxytocin, 5-HT, GABA-related signaling, and neuroimmune markers are needed to construct a more precise mechanistic network for MNPs-induced gut-brain axis toxicity.

### 4.3. Disruptive Effects of MPs and NPs on the Gut-Kidney Axis and Their Mechanisms

Compared with the gut-liver and gut-brain axes, research on the gut-kidney axis in MNPs toxicity started relatively late, and many mechanistic findings are derived from co-exposure systems. Nevertheless, notable progress has been made in the past two years [82]. The kidney is highly perfused and may be directly exposed to small particles, soluble contaminants, and gut-derived mediators circulating in the bloodstream [83]. In addition, renal function is closely dependent on systemic inflammatory and metabolic homeostasis. Therefore, the kidney is particularly vulnerable to intestinal barrier dysfunction, microbiota dysbiosis, abnormal metabolites, and circulating inflammatory signals. Previous reviews have suggested that MNPs can induce renal oxidative stress, endoplasmic reticulum stress, inflammatory responses, lipid metabolic disorders, and fibrotic remodeling, while also proposing possible renal fate pathways involving glomerular filtration and tubular reabsorption [82].

One of the most mechanistically in-depth studies using a co-exposure model of polystyrene MPs and benzo[a]pyrene (BaP) revealed that, after oral exposure, mice exhibited increased serum levels of LPS, DAO, and FITC, indicating a significant increase in intestinal permeability [84]. Meanwhile, the expression of occludin, claudin-1, and ZO-1 was downregulated, and intestinal villi were damaged, suggesting disruption of intestinal barrier integrity. Microbiota and metabolomic analyses further showed that intestinal microecological disorder was accompanied by enhanced lipid peroxidation and increased levels of polyunsaturated fatty acids such as arachidonic acid. These gut-derived abnormal metabolites were proposed to reach the kidney through the gut-kidney axis and contribute to renal injury [84]. The most important contribution of this study is that it revealed a mechanism of metabolism-mediated ferroptosis along the gut-kidney axis [84]. However, it is worth noting that the weaker ferroptotic phenotype observed *in vitro* is consistent with, but does not by itself prove, an indirect contribution of gut-derived metabolites and microbiota. The gut-kidney axis therefore represents a plausible explanation for renal injury after oral co-exposure. Because the key study used PS MPs together with BaP and did not include metabolite-rescue or microbiota-transplantation experiments, the proposed PS MPs–intestinal metabolic disturbance–renal ferroptosis sequence should be considered mechanistically suggestive rather than causally established. Moreover, gut-derived abnormal lipid metabolism could activate ACSL4/LPCAT3-related pathways in the kidney, inducing lipid peroxidation, iron homeostasis disruption, and renal ferroptosis. In contrast, *in vitro* experiments suggested that PS MPs and BaP exposure did not necessarily reproduce the same degree of ferroptotic phenotype in renal cells as observed *in vivo*. This indicates that kidney injury is largely indirectly driven by gut-derived abnormal metabolites and microbiota alterations. In other words, the gut-kidney axis provides a key explanation for why oral exposure can lead to severe renal injury. However, the core mechanism of metabolism-mediated renal ferroptosis is established based on PS MPs and BaP co-exposure systems, which has inherent limitations in interpreting pure MNPs nephrotoxicity. It remains unclear whether the intestinal lipid metabolic disorder and subsequent renal ferroptosis are triggered by PS MPs, BaP, or their synergistic toxic effects, and no current study has set rigorous single-factor control groups to eliminate pollutant interference. Additionally, the discrepancy between *in vivo* and *in vitro* ferroptotic phenotypes strongly confirms the critical role of gut-derived mediators, but existing research fails to conduct targeted intervention verification on key lipid metabolites (e.g., arachidonic acid) and gut microbiota. There is a lack of metabolite rescue or microbiota transplantation experiments to directly prove the causal chain of MNPs exposure–intestinal metabolic disorder–renal ferroptosis, and the current conclusion is only based on correlative omics evidence rather than definitive functional validation.

Complement activation has also been implicated as a key pathway linking intestinal dysfunction to renal damage. Liang et al. exposed mice to PS MPs for 8 weeks and observed intestinal barrier disruption accompanied by renal injury, as evidenced by significantly increased creatinine clearance, blood urea nitrogen (BUN), and uric acid (UA) levels [85]. Restoration of intestinal barrier integrity with antibiotics markedly alleviated PS MPs-induced kidney damage, supporting the involvement of the gut-kidney axis in MNPs-associated nephrotoxicity [85]. In addition, treatment with PMX53, a C5a receptor (C5aR) inhibitor, attenuated renal injury and reduced the expression of kidney inflammation-related markers, including MCP-1, TGF-β1, and VEGF. These results reveal that PS MPs couple intestinal dysfunction with renal damage via activation of the C5a/C5aR signaling cascade, underscoring this complement pathway as a pivotal mechanistic hub bridging the gut–kidney axis [85]. A plausible mechanism is that MNPs-induced intestinal barrier disruption enhances bacterial component and LPS translocation, thereby activating systemic complement cascades and increasing circulating C5a levels. The kidneys possess abundant blood perfusion and constitutively high C5aR expression; thus, elevated circulating C5a readily binds to renal C5aR and initiates downstream pro-inflammatory and profibrotic signaling. Such cascade events drive the recruitment and overexpression of key renal injury mediators, such as TGF-β, MCP-1, and VEGF. These findings indicate that PS MPs may connect intestinal injury with renal pathological responses through activation of the C5a/C5aR signaling cascade. The PMX53 intervention supports involvement of the C5a/C5aR pathway in this PS MP mouse model; however, it does not establish this pathway as a universal mechanism across polymers, exposure conditions, or species.

**Figure 4:**
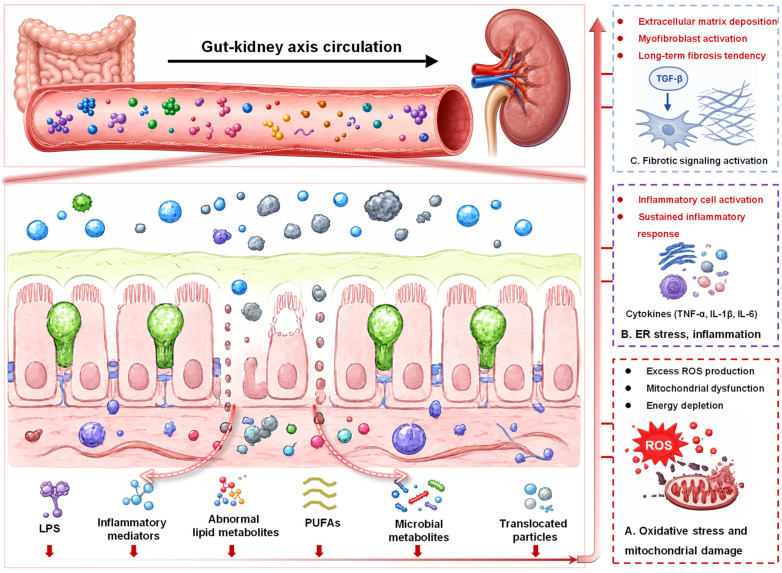
MPs/NPs disrupt the gut-kidney axis. After oral exposure, MNPs impair intestinal barrier integrity by increasing permeability, damaging villi, and downregulating tight junction proteins, including occludin, claudin-1, and ZO-1. Gut microbiota dysbiosis and lipid metabolic disturbance further promote the release of LPS, inflammatory mediators, abnormal lipid metabolites, PUFAs, microbial metabolites, and translocated particles into systemic circulation. These gut-derived signals may reach the kidney and trigger oxidative stress, mitochondrial dysfunction, endoplasmic reticulum stress, inflammation, ferroptosis, and fibrotic signaling, ultimately contributing to renal injury and fibrosis tendency. The figure was created with BioRender, PowerPoint and Adobe Illustrator.

From an integrated mechanistic perspective, MNPs-induced disruption of the gut-kidney axis involves at least four steps, as illustrated in Figure 4. First, MNPs impair intestinal barrier integrity by disrupting tight junction proteins and damaging the intestinal epithelium, leading to a “leaky gut” phenotype. Second, gut microbiota dysbiosis and altered microbial metabolism promote inflammatory activation and lipid metabolic imbalance. Third, LPS, inflammatory mediators, abnormal lipid metabolites, PUFAs, and other gut-derived signals enter systemic circulation and continuously stimulate renal tissue. Fourth, these signals induce oxidative stress, mitochondrial dysfunction, endoplasmic reticulum stress, ferroptosis, inflammatory responses, and fibrotic signaling in the kidney. Although studies directly addressing the gut-kidney axis under low-dose chronic exposure to MNPs alone remain limited, this mechanistic framework is becoming increasingly clear.

It should be emphasized that current evidence for the MNPs-related gut-kidney axis is still largely derived from combined-exposure models. This limitation should be interpreted cautiously, but it also reflects realistic environmental conditions, in which MNPs frequently coexist with polycyclic aromatic hydrocarbons, per- and polyfluoroalkyl substances, heavy metals, and other contaminants. In drinking-water systems and food chains, MNPs may act as carriers that enhance the absorption, transport, and tissue distribution of coexisting pollutants, thereby amplifying nephrotoxicity. Future studies should therefore combine single-particle exposure models with environmentally relevant mixed-exposure systems, while using fecal microbiota transplantation, barrier-repair interventions, metabolite supplementation or blockade, and pathway-specific inhibitors to verify the causal necessity and sufficiency of gut-derived signals in MNPs-induced kidney injury.

### 4.4. Disruptive Effects of MPs and NPs on the Gut-Lung Axis and Their Mechanisms

The gut-lung axis refers to a bidirectional regulatory network between the intestine and the lung mediated by microbial communities, metabolites, immune cells, inflammatory factors, and the circulatory system [86]. In recent years, the gut-lung axis has been increasingly used to explain the occurrence and progression of asthma, chronic obstructive pulmonary disease, pulmonary fibrosis, lung infections, and allergic airway inflammation [87]. Its core mechanism lies in the fact that the gut microbiota and its metabolites, such as SCFAs, tryptophan metabolites, bile acid derivatives, and lipid metabolites, can influence pulmonary immune homeostasis through the bloodstream [88]. In comparison, intestinal barrier leakage allows LPS, bacterial constituents, and pro-inflammatory mediators to disseminate into systemic circulation, which exacerbates pulmonary inflammation and disrupts lung barrier homeostasis. As such, the gut-lung axis offers an innovative systems toxicology paradigm to decipher MNPs-elicited respiratory toxicity [49].

From an exposure perspective, MNPs-induced pulmonary insults arise *via* two major routes: direct inhalation, and gut-mediated indirect secondary effects following oral exposure. Inhaled MNPs deposit directly within the airway and alveolar regions, triggering localized oxidative stress, compromising epithelial barrier integrity, remodeling lung microbial communities, and initiating robust inflammatory cascades. In contrast, orally ingested MNPs first act on the intestine, causing gut microbiota dysbiosis, intestinal mucosal injury, and metabolic abnormalities, and subsequently affect the lung through the circulatory system [89]. This means that MNPs-induced pulmonary toxicity should not be understood solely as the result of “particles directly entering the lung”; the indirect injury chain of “intestinal initiation–circulatory transport–pulmonary response” should also be considered [90]. Recent reviews on airborne MNPs have suggested that airborne MPs and NPs have become a potential source of respiratory health risks [91], while systematic reviews on lung health have also shown that MNPs are associated with pulmonary cytotoxicity, oxidative stress, mitochondrial damage, immune inflammation, and potential risks of chronic lung diseases [92].

Although experimental studies directly focusing on MNPs and the gut-lung axis are still at an early stage, several key studies have provided relatively strong evidence. Xuan et al. found that exposure to 50-100 nm NPs could simultaneously induce lung injury, intestinal mucosal damage, and gut microbiota dysbiosis in mice [49]. Among these changes, gut microbiota-derived lactate accumulated in the intestine, serum, and lung tissues and promoted epithelial-mesenchymal transition through the HIF1α/PTBP1 pathway, thereby aggravating lung injury. By using germ-free mice, antibiotic-mediated microbiota depletion, and fecal microbiota transplantation, this study provided relatively direct evidence for a causal link between gut microbiota-derived metabolites and lung injury. Kaluç et al. further found that oral ingestion of PET NPs caused alterations in both colonic and pulmonary microbiota, characterized by an increase in Gram-negative bacteria and a decrease in *Lactobacillus* in the colon, as well as increased *Pseudomonas* abundance and abnormal microbial energy metabolism in the lung [93]. These findings suggest that oral NPs exposure may affect respiratory health through the gut-lung axis.

Mechanistically, the disruption of the gut-lung axis by MNPs can be broadly summarized into three pathways. The first is the “intestinal barrier disruption–LPS translocation–pulmonary inflammation” pathway. MNPs can disrupt intestinal epithelial tight junctions and increase intestinal permeability, allowing LPS and bacterial components to more readily enter the circulatory system, where they can activate inflammatory pathways such as TLR4/NF-κB signaling and the NLRP3 inflammasome in the lung. Zeng et al., in a study on PS NP-exacerbated asthma, further proposed that gut microbiota dysbiosis can release more hexa-acylated LPS, activate the intestinal TLR4/NF-κB pathway, and promote pulmonary inflammatory responses through the gut-lung axis [45]. This study also found that microbiota dysbiosis led to decreased SCFAs and abnormal glycerophospholipid and amino acid metabolism, thereby enhancing PLA2 activity in lung tissue and forming a PLA2-TRPV1 neuroimmune positive feedback loop, ultimately aggravating airway hyperresponsiveness and lung injury. One proposed route links intestinal barrier impairment with increased systemic exposure to LPS and other gut-derived inflammatory mediators, which may contribute to pulmonary inflammation in susceptible experimental models. However, this sequence has not been established as necessary or sufficient, and direct particle effects, lung-resident microbiota, and other circulating mediators may act in parallel.

The second pathway is the abnormal microbial metabolites–pulmonary immune remodeling pathway. SCFAs, lactate, tryptophan metabolites, bile acid derivatives, and lipid metabolites produced by the gut microbiota can regulate the functions of pulmonary macrophages, dendritic cells, T cells, and epithelial cells. After MNPs exposure, alterations in the gut microbiota metabolic profile may reduce anti-inflammatory metabolites or increase pro-inflammatory metabolites, thereby disrupting pulmonary immune homeostasis. Wu et al. further indicated that chronic NPs exposure can disturb tryptophan metabolic homeostasis in the gut-lung-microbiota axis and induce chronic toxicity in intestinal and lung tissues [89], suggesting that tryptophan metabolism and its downstream immunoregulatory pathways may represent important nodes in MNPs-mediated gut-lung axis injury.

The third pathway is the “local lung microbiota alteration–amplification of inflammation and ferroptosis/fibrosis” pathway [94]. In addition to the gut microbiota, the lung itself harbors a low-abundance but functionally important microbial community [95]. Inhaled PS MPs can alter the structure of the lung microbiota, increase Gram-negative bacteria, promote LPS release, and induce ferroptosis in lung tissue through TLR4-mediated iron homeostasis imbalance, ultimately leading to impaired lung function. Fibrous MPs can also exacerbate ovalbumin-induced asthma responses by altering lung microbiota composition. In addition, PS MPs can induce lung injury through TLR2/NF-κB signaling and may promote pulmonary fibrosis through cGAS/STING activation and ferroptosis in alveolar epithelial cells [96]. These findings indicate that lung microbiota dysbiosis may interact with gut-derived signals and local particle deposition to amplify pulmonary inflammation and tissue remodeling.

**Figure 5:**
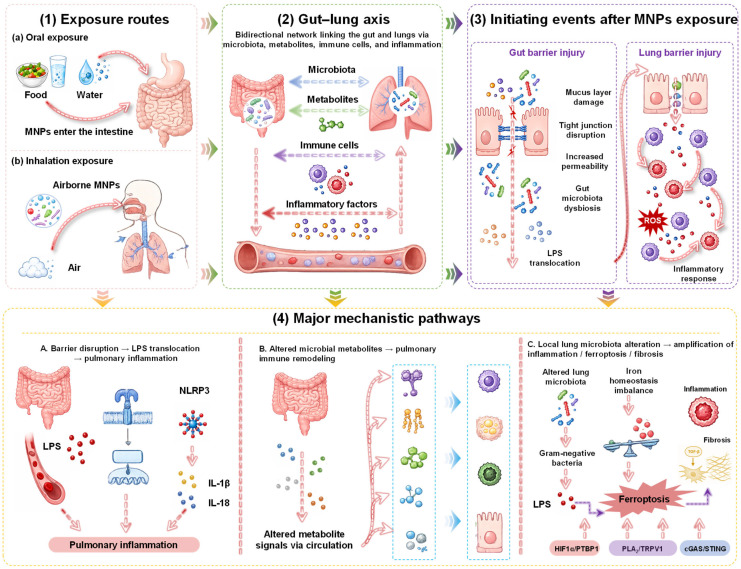
MPs/NPs disrupt the gut-lung axis. MNPs-induced pulmonary toxicity may occur through direct inhalation or gut-mediated indirect pathways after oral exposure. Inhaled MNPs can directly impair the airway and alveolar barriers, alter lung microbiota, and induce local oxidative stress and inflammation. Orally ingested MNPs can disrupt intestinal barrier integrity, reshape gut microbiota, and alter circulating metabolites and inflammatory mediators. These gut-derived signals, including LPS, SCFAs, lactate, tryptophan metabolites, lipid metabolites, and bile acid derivatives, may reach the lung and activate TLR4/NF-κB, NLRP3, HIF1α/PTBP1, PLA2/TRPV1, cGAS/STING, ferroptosis, and fibrotic signaling, ultimately contributing to pulmonary inflammation, airway hyperresponsiveness, and fibrosis tendency. The figure was created with BioRender, PowerPoint and Adobe Illustrator.

Overall, the disruption of the gut-lung axis by MNPs can be summarized as follows (Figure 5): after oral or inhalation exposure, MNPs impair intestinal and/or pulmonary barriers, induce dysbiosis of both gut and lung microbiota, alter key signaling molecules such as lactate, short-chain fatty acids, tryptophan metabolites, lipid metabolites, and LPS, and amplify pulmonary inflammation, airway hyperresponsiveness, and fibrosis risk through pathways including TLR4/NF-κB, NLRP3, HIF1α/PTBP1, PLA2/TRPV1, cGAS/STING, and ferroptosis. However, compared with the gut-liver and gut-brain axes, research on the MNPs-related gut-lung axis remains at an early stage. Future investigations are warranted to further distinguish direct pulmonary toxicity induced by inhalation from gut-derived indirect adverse effects. Integrated strategies incorporating germ-free animal models, fecal microbiota transplantation, lung microbiota modulation, metabolite supplementation and neutralization, as well as lung organoid platforms, should be adopted to systematically verify the causal necessity and sufficiency of the gut microbiota-metabolite-lung injury regulatory axis. Nevertheless, this inference remains model-specific and does not exclude parallel contributions from direct particle translocation, other circulating mediators, or lung-resident microbiota. Other proposed gut-lung pathways remain largely preclinical and have not generally been established as necessary and sufficient.

### 4.5. Disruptive Effects of MPs and NPs on the Gut-Reproductive Axis and Gut–Mammary Axis

The reproductive system is another central research frontier in MNPs toxicological studies [9,97]. Accumulating evidence indicates that MNPs exert detrimental impacts on ovarian and testicular integrity, as well as gamete quality in both sexes, via the induction of oxidative stress, inflammation, hormonal dyshomeostasis, apoptosis, and ferroptosis [98,99]. However, from the perspective of gut-organ axis biology, mechanistic evidence for the gut-reproductive axis remains insufficient. Most existing studies have mainly reported associations between gut microbiota dysbiosis and reproductive impairment, whereas rigorous causal frameworks comparable to those established for the gut-liver axis have not yet been fully developed.

Nevertheless, the regulatory role of the gut microbiota in reproductive health has attracted increasing attention [100,101]. Evidence from gut-testis axis studies have shown that the intestinal microecology participates in spermatogenesis and the maintenance of the testicular microenvironment through endocrine, immune, and metabolic regulation. Since MNPs themselves can induce microbiota dysbiosis, chronic low-grade inflammation, and endocrine disruption, it can be theoretically inferred that their reproductive toxicity is unlikely to be entirely independent of the intestine [84,101]. Instead, it may be partly mediated by systemic inflammation and metabolic reprogramming caused by gut microbiota and barrier abnormalities [84]. Therefore, the gut-reproductive axis should currently be regarded as an emerging mechanistic hypothesis that requires further causal validation.

Evidence supporting a potential gut-ovary axis has begun to appear. Zhang et al., investigated the effects of PS MPs and PLA MPs on ovarian reserve function and follicular development in female mice [102]. *In vitro* experiments showed that PLA MPs exhibited stronger cytotoxicity toward granulosa cells, whereas in vivo experiments found that PS MPs caused more pronounced damage to the intestinal barrier and ovarian reserve function. Both types of MPs disrupted the estrous cycle, reduced the number of follicles at different developmental stages, decreased anti-Müllerian hormone (AMH) and estradiol (E2) levels, and induced uterine tissue injury. The study suggests that PS MPs may indirectly exacerbate ovarian functional decline by disrupting the intestinal barrier, whereas PLA MPs may exert more direct toxicity on granulosa cells, the largest cell population within ovarian follicles [102]. Therefore, environmental MPs may interfere with female reproductive function through both direct gonadal toxicity and indirect gut-derived pathways. However, direct causal evidence demonstrating that gut microbiota or specific gut-derived metabolites are necessary and sufficient for MPs-induced reproductive injury remains limited.

Compared with the gut-reproductive axis, more direct evidence has emerged for the gut-mammary axis in MNPs research [48]. A recent study showed that oral exposure to commonly encountered PS NPs markedly accelerated the development and metastasis of triple-negative breast cancer, the most aggressive subtype of breast cancer, by reshaping the gut microbiota, increasing blood glutamate levels, and activating platelets [103]. This study revealed, for the first time, a complete “gut microbiota–metabolite–platelet” axis linking environmental exposure to cancer progression, offering a new perspective for understanding the carcinogenic mechanisms of pollutants [103]. Similarly, 1 μm PS MPs, under lactational exposure conditions, could disrupt the intestinal barrier, induce colonic inflammation and gut microbiota dysbiosis, and were also detected in mammary tissue, where they caused mammary inflammation, lipid metabolic disorder, and ferroptosis [103]. Importantly, fecal microbiota transplantation reproduced the phenotypes of blood–milk barrier leakage and mammary inflammation, directly demonstrating the mediating role of gut microbiota in mammary injury. However, it is critical to acknowledge that the entirety of these notable research findings was obtained solely through investigations carried out on animal subjects rather than human participants. Owing to inherent biological discrepancies in metabolic pathways, physiological regulatory systems, and exposure response thresholds between laboratory animals and human populations, the reliable transferability of the animal-based risk assessment results to accurately quantify corresponding human health hazards remains highly uncertain and cannot be assumed without additional human clinical or epidemiological evidence.

Another multi-organ crosstalk study further proposed the concept of the “gut-liver-mammary axis” [48]. This study found that PS MPs mainly accumulated in feces, colon, and liver in maternal mice, but could also affect mammary tissue. Both the intestinal barrier and the blood–milk barrier were disrupted, and inflammation and pathological injury occurred in the liver, intestine, and mammary gland. Metabolomic and metagenomic analyses showed that abnormal hepatic bile acid metabolism and gut microbiota alterations jointly participated in this cross-organ injury process. Moreover, depletion of the gut microbiota markedly alleviated tissue inflammation and barrier leakage. These findings indicate that MNPs-induced distal organ injury may not occur through a simple unidirectional distribution pathway, but may instead involve network-like toxicity mediated by sequential crosstalk among the gut, liver, and mammary gland.

**Figure 6:**
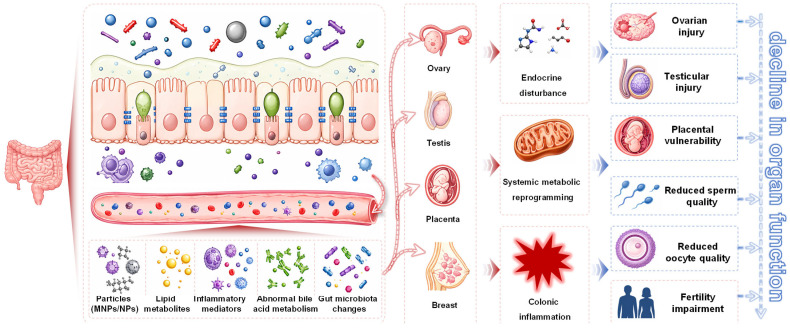
MPs/NPs disrupt the gut-reproductive and gut-mammary axes. MNPs exposure may induce gut microbiota dysbiosis, intestinal barrier dysfunction, chronic low-grade inflammation, endocrine disturbance, and systemic metabolic reprogramming. For the gut-reproductive axis, current evidence suggests potential effects on ovarian, testicular, placental, and gamete health, but causal gut-derived mechanisms remain incompletely validated. In contrast, the gut–mammary axis has stronger experimental support, including evidence from fecal microbiota transplantation and gut-liver-mammary crosstalk. The figure was created with BioRender, PowerPoint and Adobe Illustrator.

Overall, current evidence suggests that the gut-mammary axis has progressed from a conceptual framework toward experimental validation (Figure 6), whereas the gut-reproductive axis remains in a transitional stage between theoretical hypothesis and mechanistic confirmation. Future studies should combine fecal microbiota transplantation, germ-free animal models, isotope tracing, single-cell sequencing, metabolomics, and hormonome analysis to clarify whether gut-derived inflammatory, metabolic, and endocrine signals are causally involved in MNPs-induced injury to the ovary, testis, placenta, and mammary gland. Such studies will be essential for distinguishing direct particle toxicity from indirect gut-mediated reproductive and mammary effects. Moreover, MNPs exposure can coincide with intestinal dysbiosis, chronic low-grade inflammation, metabolic disturbance, and endocrine disruption, a gut-mediated contribution to reproductive injury is biologically plausible. However, plausibility should not be interpreted as evidence of necessity or sufficiency. Current evidence is predominantly animal-based and associative, and the gut-reproductive axis should therefore be regarded as a hypothesis-generating framework pending fecal microbiota transplantation, germ-free, metabolite-rescue, barrier-repair, and pathway-blockade studies.

### 4.6. Other Potential Gut-Organ Axes: From the Gut-Skin and Gut-Heart Axes to the Gut-Immune Network

In addition to the axes discussed above, an increasing number of reviews have begun to place MNPs toxicity within a broader framework of the “gut-distal organ network”. This concept emphasizes that intestinal homeostasis may influence multiple remote tissues through microbial metabolites, immune mediators, circulating inflammatory signals, and barrier-derived endotoxin translocation [104,105], immune dysregulation [46], cardiometabolic diseases [106,107,108], gut microbiota-hypoxanthine-Wnt axis-related outcomes [109]. These observations imply that the toxicological impact of MNPs may extend beyond isolated organ injury and may instead involve system-level pathological cascades initiated by intestinal disruption. For the skin, cardiovascular system, and immune network, several upstream events may be shared with the major gut-organ axes described earlier. Gut microbiota dysbiosis, increased intestinal permeability, LPS translocation, abnormal microbial metabolites, and chronic low-grade inflammation may provide common biological signals linking intestinal injury to distal tissue responses. In this context, MNPs-induced systemic toxicity may involve not only direct particle distribution to target organs, but also indirect gut-derived regulation of immune tone, metabolic balance, vascular homeostasis, and epithelial barrier function.

However, the evidence supporting these extended axes remains considerably weaker than that for the gut-liver, gut-brain, and gut-kidney axes. Although vascular injury, immune imbalance, skin-related alterations, and cardiometabolic disturbances have been observed in some studies, it has not yet been fully established whether these outcomes are primarily driven by gut-mediated mechanisms, direct particle translocation, co-contaminant toxicity, or generalized systemic inflammation. Therefore, these extended axes should be regarded as hypothesis-generating directions rather than fully established mechanistic frameworks. Future studies should use fecal microbiota transplantation, germ-free animal models, metabolite supplementation or blockade, barrier-repair strategies, and tissue tracing approaches to determine the causal contribution of gut-derived signals to MNPs-induced skin, cardiovascular, and immune toxicity.

## 5. A Unified Four-Layer Model of MNPs-Induced Gut–Organ Axis Toxicity

**Figure 7:**
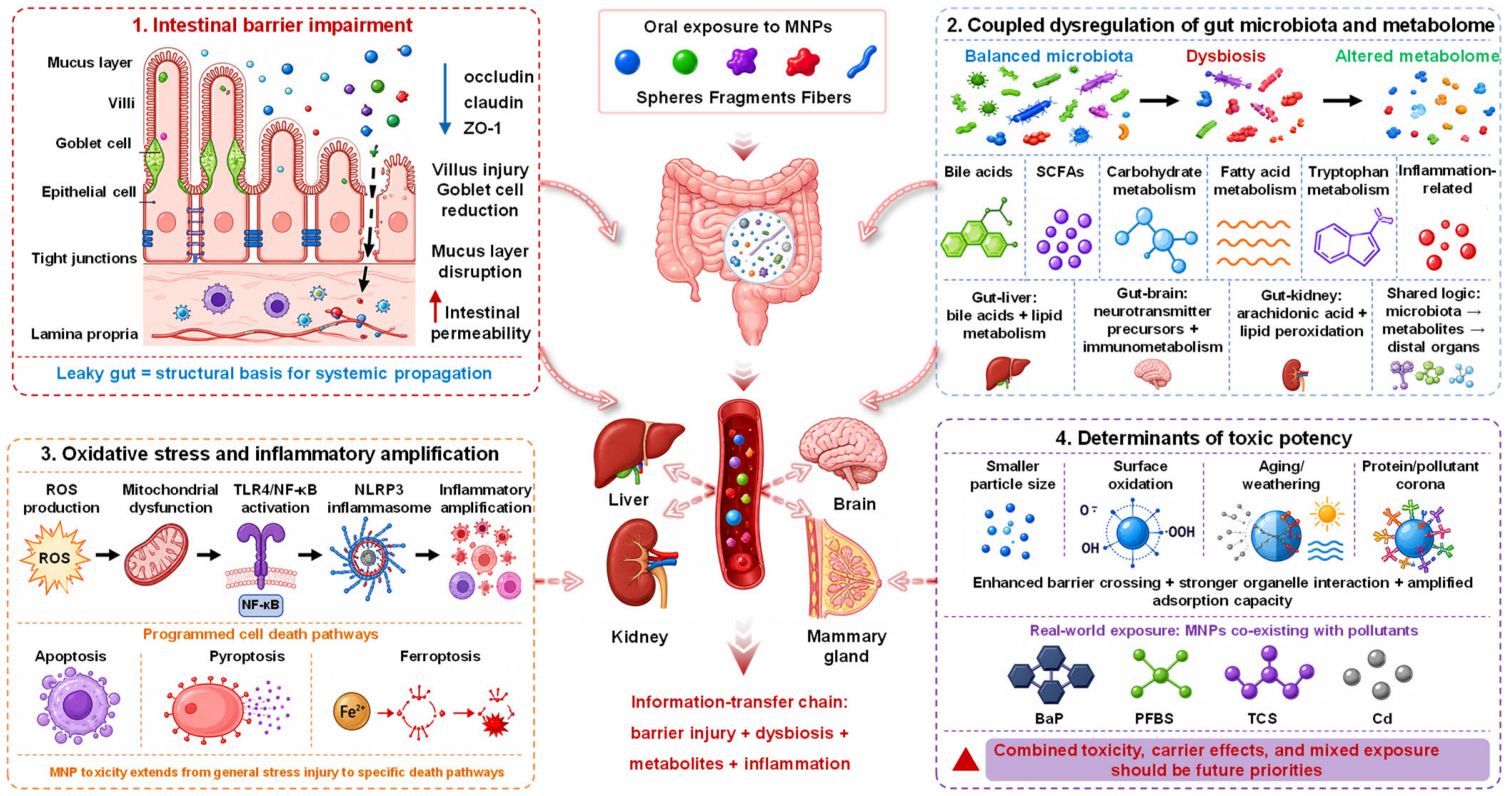
A unified four-layer model of MNPs-induced gut-organ axis toxicity. Oral exposure to MNPs first induces particle-barrier interactions, leading to mucus disruption, tight junction downregulation, villus injury, goblet cell loss, and increased intestinal permeability. Barrier dysfunction is accompanied by gut microbiota dysbiosis and metabolite remodeling, involving bile acids, SCFAs, fatty acids, tryptophan metabolites, uric acid, and inflammation-related metabolites. These gut-derived signals further activate oxidative stress, mitochondrial dysfunction, TLR4/NF-κB signaling, NLRP3 inflammasome responses, and programmed cell death, including apoptosis, pyroptosis, and ferroptosis, in distal organs. Particle size, morphology, surface chemistry, aging, bio-corona/plastisphere formation, and co-contaminant adsorption further modulate the intensity and specificity of multi-organ toxicity. The figure was created with BioRender, PowerPoint and Adobe Illustrator.

Collectively, evidence from different gut-organ axes suggest that MNPs-induced systemic toxicity can be interpreted through a unified four-layer mechanistic model, as illustrated in Figure 7. The first layer is particle-barrier interaction. Following oral exposure, MNPs initially interact with the intestinal mucus layer, epithelial cells, immune components, and resident microbiota. Across the gut-liver, gut-brain, gut-kidney, gut-lung, and gut-mammary axes, intestinal barrier dysfunction is one of the most consistently observed initiating events. This process is characterized by the downregulation of tight junction proteins, including occludin, claudin-1, and ZO-1, together with villus injury, goblet cell loss, mucus layer disruption, and increased intestinal permeability. These alterations contribute to the formation of a “leaky gut” phenotype, which provides a structural basis for the systemic dissemination of gut-derived toxic signals.

The second layer is microbiota-metabolite remodeling. MNPs-induced gut microbiota dysbiosis is not merely reflected by changes in microbial abundance or diversity, but also by functional reprogramming of microbial metabolism. Altered gut microbiota can reshape bile acid metabolism, short-chain fatty acid production, carbohydrate utilization, fatty acid metabolism, tryptophan/kynurenine pathways, and inflammation-related metabolites. The dominant mediators may differ among specific axes. For example, the gut-liver axis is closely associated with bile acid dysregulation, uric acid accumulation, and lipid metabolic disorder; the gut-brain axis is more strongly linked to neurotransmitter precursors, SCFAs, tryptophan metabolites, and immunometabolites; whereas the gut-kidney axis highlights arachidonic acid metabolism, lipid peroxidation, and ferroptosis-related metabolites. Despite these differences, these axes converge on a common information-transmission chain involving gut microbiota, abnormal metabolites, and distal organ injury.

The third layer comprises immune-inflammatory amplification and programmed cell death. MNPs-induced barrier disruption and microbial dysbiosis can facilitate the translocation of lipopolysaccharide, bacterial components, inflammatory mediators, and abnormal metabolites into systemic circulation. These signals may activate oxidative stress, mitochondrial dysfunction, TLR4/NF-κB signaling, NLRP3 inflammasome responses, and other inflammatory pathways in distal organs. As the field has advanced, the understanding of MNPs toxicity has extended beyond general oxidative injury and apoptosis to more specific forms of programmed cell death, including pyroptosis and ferroptosis. For instance, NPs have been associated with hepatic pyroptosis, PS and BaP co-exposure can induce renal ferroptosis through gut-derived metabolic disturbance, and PS exposure may promote mammary ferroptosis through gut-mammary axis regulation. These findings indicate that MNPs-induced gut-organ axis toxicity involves not only inflammatory signal transmission, but also organ-specific execution of cell death and tissue remodeling.

The fourth layer is modulation by particle physicochemical properties and environmental transformation. The strength and specificity of MNPs-induced gut-organ axis toxicity is strongly influenced by particle size, morphology, surface charge, surface oxidation, environmental aging, and corona formation. In general, smaller particles, especially NPs, are more likely to cross the intestinal barrier, enter systemic circulation, interact with intracellular organelles, and accumulate in distal organs. Surface oxidation and weathering can increase roughness, functional groups, and adsorption affinity, thereby modifying biological reactivity and toxic potency. In addition, protein coronas, pollutant coronas, and plastisphere formation can alter the biological identity of MNPs and affect their interactions with epithelial cells, immune cells, and microbiota. Under realistic environmental conditions, MNPs rarely exist as pristine and chemically pure particles; instead, they often coexist with contaminants such as BaP, perfluorobutanesulfonic acid, triclosan, cadmium, and other organic or inorganic pollutants. Therefore, mixed exposure, carrier effects, and environmentally transformed particles should be incorporated into future gut-organ axis studies.

## 6. Major Limitations in the Current State of Research

Although research on MNPs toxicity has expanded rapidly in recent years, the increasing number of publications has not yet translated into robust evidence certainty or quantitative risk assessment. The World Health Organization emphasized in its 2022 report that available data on dietary and inhalation exposure remain insufficient to support comprehensive human health risk assessment. Similarly, the 2026 official statement from the European Food Safety Authority (EFSA) highlighted the lack of standardized protocols for the detection, characterization, and quantification of MNPs in food matrices, leaving considerable uncertainty in health risk appraisal. These observations indicate that the field is currently in a transitional stage: hazard identification is becoming clearer, whereas exposure assessment, causal inference, and quantitative risk evaluation remain poorly established.

Human epidemiological evidence should also be interpreted with caution. A rapid systematic review classified microplastic-associated adverse outcomes in the human digestive, respiratory, and reproductive systems as suspected toxic hazards [110]. Although evidence related to digestive health has reached a moderate level of credibility, it remains insufficient to support definitive, consistent, and quantitatively validated causal conclusions. In other words, current human evidence has important preventive and warning value, but it has not yet formed a closed-loop risk assessment framework linking external exposure, internal dose, tissue burden, biological mechanism, and disease outcome.

Methodological limitations represent another major bottleneck [111,112]. Many toxicological studies still rely on high-dose, short-term exposure to a single polymer type and regular spherical particles, which differs substantially from real-world exposure characterized by low-dose, chronic, mixed, aged, and morphologically diverse particles [113,114,115]. In addition, many studies provide insufficient particle characterization, often lacking detailed information on particle size distribution, morphology, surface charge, surface oxidation, additive composition, endotoxin contamination, and corona formation [111,112]. These limitations make it difficult to compare results across studies or extrapolate experimental findings to realistic human exposure scenarios.

Analytical detection of MNPs in human tissues and biological samples remains particularly challenging. Current studies still face problems such as insufficient contamination control, inadequate blank correction, uncertain recovery rates, difficulty in excluding false positives, and poor inter-laboratory comparability (Table 3). Therefore, a substantial methodological gap remains between particle detection and disease causation. Future human biomonitoring studies should adopt plastic-free sampling procedures, procedural blanks, recovery experiments, and cross-validation using complementary techniques such as FTIR, Raman spectroscopy, pyrolysis-GC/MS, and electron microscopy. Without standardized detection and reporting criteria, evidence from human samples will remain difficult to integrate into reliable risk assessment.

Moreover, causal validation remains insufficient in gut-organ axis research. Most studies are still dominated by mouse models and endpoint phenotypes, while rigorous mechanistic verification is limited [116]. Although fecal microbiota transplantation, antibiotic-mediated microbiota depletion, and multi-omics analyses have begun to clarify the role of gut microbiota and metabolites, studies using germ-free animals, time-series tracking, single-cell-resolution analysis, multi-organ isotope tracing, metabolite supplementation or blockade, barrier-repair interventions, and intervention-reversal experiments remain scarce [7]. This limitation is particularly evident for the gut-reproductive, gut-heart, gut-skin, and gut-lung axes, where many conclusions remain inferential rather than causally demonstrated. Therefore, future studies should move beyond the simple observation that the gut changes and distal organs are also injured and instead determine whether gut-derived signals are necessary and sufficient for MNPs-induced multi-organ toxicity. Additionally, most available studies are better suited to hazard identification than to quantitative human risk assessment. Their relevance is constrained by high or bolus doses, short exposure periods, monodisperse spheres, artificial surface functionalization, limited internal dosimetry, and uncertain comparability with chronic low-level exposure in human populations.

An additional limitation is the pronounced polymer imbalance in the mechanistic literature. A large proportion of gut-organ axis studies use commercially available, spherical PS particles with narrow size distributions and, frequently, fluorescent labels. PS is experimentally convenient because particle size, surface charge, and labeling can be controlled; however, it does not represent the physicochemical diversity of environmentally encountered MNPs. Polymer backbone chemistry, density, crystallinity, hydrophobicity, additive and leachate profiles, degradability, particle shape, surface charge, environmental aging, and bio-corona formation can alter gastrointestinal persistence, microbial interactions, epithelial uptake, systemic translocation, and organ-specific toxicity. Accordingly, mechanisms established with PS particles, including specific transporter-assisted uptake, complement activation, or defined immune-metabolic pathways, should not be assumed to apply quantitatively or qualitatively to polyethylene, polypropylene, polyethylene terephthalate, polyvinyl chloride, polylactic acid, starch-based particles, fibers, fragments, or environmentally aged mixtures. Evidence from non-PS polymers suggests that some upstream events, such as barrier impairment, microbiota dysbiosis, oxidative stress, and metabolic disturbance, may recur across polymers, whereas downstream potency and dominant pathways can differ.

## 7. Conclusions and Outlooks

Overall, the biological hazards of MNPs can no longer be adequately explained by single-organ toxicity alone. Increasing evidence indicates that the intestine functions as an initiating hub for MNPs-induced systemic toxicity. Gut microbiota dysbiosis, epithelial barrier disruption, abnormal metabolite transport, immune-inflammatory activation, oxidative stress, mitochondrial dysfunction, and programmed cell death collectively constitute the major biological framework through which MNPs propagate injury along gut–organ axes. Among the currently investigated axes, the gut-liver axis is supported by the most mature mechanistic evidence, whereas the gut-brain and gut-kidney axes are becoming increasingly clear. The gut-mammary axis has begun to receive direct experimental support, while the gut-reproductive axis and other extended axes, such as the gut-skin and gut-heart axes, still require more rigorous causal validation. Therefore, MNPs-induced systemic toxicity should be understood as the result of multiple interacting mechanisms, including particle-specific effects, chemical carrier functions, microbiota-metabolite remodeling, immune-inflammatory amplification, and organ-specific pathological responses.

This conceptual framework marks an important shift in MNPs toxicology. Future studies should move beyond simply asking whether plastic particles are intrinsically toxic and should instead clarify how MNPs initiate, transmit, and amplify systemic injury through gut-associated regulatory networks. At the same time, defining realistic exposure thresholds that produce measurable biological effects remains essential for translating mechanistic findings into human health risk assessment. This transition will move the field from descriptive hazard identification toward quantitative risk evaluation, and from isolated organ pathology toward integrated systems toxicology.

Future research should first return to realistic exposure scenarios. Greater priority should be given to environmentally relevant concentrations, long-term chronic exposure designs, and exposure systems that reflect actual particle spectra in food, drinking water, and air. Studies should incorporate multiple polymer types, diverse particle morphologies, environmentally aged particles, and mixed-exposure models involving per- and polyfluoroalkyl substances, polycyclic aromatic hydrocarbons, metals, and other coexisting contaminants. Such designs are necessary to ensure that experimental findings are applicable to population-level risk assessment rather than remaining limited to proof-of-toxicity models.

Second, causal validation should become a core criterion for gut-organ axis research. Future studies should not merely report that intestinal changes and distal organ injuries occur simultaneously. Instead, they should determine whether gut-derived mediators are necessary and sufficient for MNPs-induced organ toxicity. Germ-free animals, fecal microbiota transplantation, colonization experiments, key metabolite supplementation or blockade, intestinal barrier-repair interventions, pathway-specific inhibitors, and organoid co-culture systems should be used to verify the roles of gut microbiota, LPS, bile acids, short-chain fatty acids, tryptophan metabolites, lipid metabolites, and other candidate mediators. Only through such approaches can correlative observations be upgraded to mechanistic evidence.

Third, standardized analytical detection and data reporting should be strengthened. Reliable human biomonitoring and cross-study comparison require plastic-free procedures during sample collection and processing, procedural blank controls, recovery rate reporting, and cross-validation using complementary techniques such as Fourier-transform infrared spectroscopy, Raman spectroscopy, pyrolysis-gas chromatography/mass spectrometry, and electron microscopy. In addition, particle size distribution, morphology, polymer type, surface chemistry, aging status, and potential contamination should be reported in a unified manner. Without analytical standardization and quality control systems across laboratories, studies on MNPs exposure and gut-organ axis toxicity will remain difficult to integrate into a coherent evidence network.

Finally, future research should extend from damage mechanisms to intervention strategies. A limited number of studies have suggested that regulating gut microbiota, repairing intestinal barrier function, improving bile acid metabolism, and inhibiting lipid peroxidation may partially alleviate MNPs-induced distal organ injury. Further systematic validation is needed for probiotics, prebiotics, postbiotics, dietary fiber, antioxidant nutrients, bile acid modulators, ferroptosis inhibitors, and barrier-protective factors. Nevertheless, this direction remains largely based on animal and mechanistic studies. Therefore, these interventions should not yet be interpreted as mature prevention or treatment recommendations for humans. Instead, they should be regarded as experimental strategies for identifying modifiable biological pathways and potential protective targets in MNPs-induced systemic toxicity.

## Figures and Tables

**Table 1:** Summary of recent reviews concerning gut-organ axis disruption induced by MPs/NPs.

No.	Topic	Main Content	Refs.
1	Gut-Brain Axis	Systematically reviews the potential coordinated effects of gastrointestinal nanoplastic exposure on the gut and brain, with emphasis on the intestinal barrier, microbiota, and neural, immune, and endocrine signaling.	[28]
2	Gut Microbiota/Ecology	Systematically evaluates the effects of microplastic exposure on gut microbiota and intestinal mucosal morphology and function across animal species, while comparing differences in polymer type, particle size, dose, and species.	[29]
3	Gut Microbiota/Ecology	Reviews the bidirectional interactions between micro-/nanoplastics and the gut microbiota, including dysbiosis, microbial metabolic responses, and the potential for plastic biotransformation or biodegradation.	[30]
4	Intestinal Barrier/GI Toxicity	Uses an adverse outcome pathway framework to assess gastrointestinal hazards of orally ingested microplastics, from molecular initiating events through cellular, tissue, and organ-level effects.	[31]
5	Gut-Liver Axis	Summarizes nanoplastic accumulation, tissue injury, and metabolic abnormalities in the liver, while also addressing pathways through which intestinal injury and microbiota alterations may enhance hepatic exposure.	[32]
6	Gut-Brain Axis	Uses oral exposure as the central pathway to connect intestinal accumulation, tissue injury, immune activation, and microbiota alterations with cerebral and systemic outcomes.	[33]
7	Gut-Liver Axis	Explains microplastic-induced liver injury specifically through the gut-liver axis: after changes in the gut microbiota and barrier, microbial products and metabolites reach and influence the liver through the portal circulation.	[34]
8	Gut-Liver Axis	Summarizes micro-/nanoplastic exposure, circulatory translocation, and hepatic accumulation, integrating direct hepatotoxicity with indirect effects related to the gut-liver axis.	[35]
9	Gut Microbiota/Ecology	Focuses on the human gut microbiome and discusses possible links among microplastic exposure, dysbiosis, intestinal inflammation, and multiple chronic diseases.	[36]
10	Gut-Brain Axis	Integrates pathways of micro-/nanoplastic-induced brain injury around the microbiota-gut-brain axis and discusses the gut microbiota as a potential intervention target.	[37]
11	Gut-Brain Axis	Systematically links micro-/nanoplastic-induced gut dysbiosis, increased intestinal permeability, and systemic inflammation with neurodegenerative changes in the brain.	[38]
12	Gut-Brain Axis	Summarizes neurodevelopmental, neurobehavioral, and neurodegenerative abnormalities associated with micro-/nanoplastics and identifies the gut-brain axis as an important indirect mechanism.	[39]
13	Multi-Organ Axes	Uses organ axes as an organizing framework to integrate seven propagation pathways: gut-liver, gut-brain, gut-endocrine, liver-kidney, HPA, HPG, and placenta-fetus axes.	[40]
14	Gut-Liver Axis	Uses the plastic-gut-liver axis as the central framework to integrate exposure, intestinal uptake, systemic distribution, inflammatory and metabolic reprogramming in the liver, and pancreatic/β-cell stress.	[41]
15	Gut-Brain Axis	Systematically reviews micro-/nanoplastic exposure, intestinal accumulation, and neural, immune, and endocrine transmission pathways along the microbiota-gut-brain axis.	[42]

**Table 2:** Literature search strategy.

Item	Search Strategy
Databases	Web of Science, PubMed, Scopus, and Google Scholar
Search period	The search window is now specified as 2021 to 2026.
Search terms	Terms include microplastics, nanoplastics, gut-organ axis, gut-liver axis, gut-brain axis, gut-kidney axis, gut-lung axis, gut microbiota, intestinal barrier, metabolomics, oxidative stress, inflammation, ferroptosis, pyroptosis, and related terms.
Document types	Peer-reviewed original research articles and mechanistic reviews were considered. Book chapters, theses, conference abstracts, and non-biological occurrence-only studies were excluded from evidence grading.
Inclusion criteria	Studies reporting particle characterization, intestinal barrier dysfunction, gut microbiota changes, metabolomic alterations, distal organ injury, or causal validation were included.

**Table 3:** Analysis methods for MPs/NPs and their advantages and disadvantages.

Technique/Device	Information Obtained	Main Strengths	Main Limitations
Optical microscopy/stereomicroscopy	Particle count, shape, color, approximate size for larger MPs	Rapid screening and visual inspection	Cannot reliably identify polymer type; poor performance for small MPs and NPs
µ-FTIR/FTIR imaging	Polymer identity, particle distribution, approximate size for MPs	Non-destructive polymer identification; useful for food and tissue samples	Spatial resolution limits detection of small MPs/NPs; background contamination must be controlled
Raman/micro-Raman spectroscopy	Polymer identity and chemical signatures at smaller particle sizes	Higher spatial resolution than FTIR; useful for small particles	Fluorescence interference, long acquisition time, and risk of sample heating
Pyrolysis-GC/MS or TED-GC/MS	Polymer mass, additives, and plastic-associated chemicals	Sensitive quantitative mass-based detection	Destructive; does not provide particle number, shape, or size distribution
SEM/TEM/AFM	Nanoscale morphology, surface structure, aggregation, and particle–cell interface	High-resolution visualization of small particles and biointerfaces	Limited polymer identification unless coupled with chemical analysis; sample preparation artifacts are possible
DLS/NTA	Hydrodynamic size distribution and particle number in suspension	Useful for engineered NP suspensions and aggregation behavior	Affected by aggregation and biological matrices; cannot identify polymer type
XPS/ToF-SIMS/zeta potential	Surface chemistry, oxidation, charge, and corona-related surface changes	Useful for assessing aging, oxidation, and biological identity	Limited direct applicability to complex tissue matrices; often needs complementary methods

## Data Availability

No new data were created or analyzed in this study. Data sharing is not applicable to this article.
